# Evaluation of the First Contact Physiotherapy (FCP) model of primary care: a qualitative insight

**DOI:** 10.1016/j.physio.2021.08.003

**Published:** 2021-12

**Authors:** R. Goodwin, F. Moffatt, P. Hendrick, S. Stynes, A. Bishop, P. Logan

**Affiliations:** a Division of Rehabilitation, Ageing and Wellbeing, Faculty of Medicine & Health Sciences, University of Nottingham, United Kingdom; b Physiotherapy and Sport Rehabilitation, Faculty of Medicine & Health Sciences, University of Nottingham, United Kingdom; c Keele University, School of Medicine, Keele, United Kingdom; d Midlands Partnership Foundation NHS Trust, Haywood Hospital Spinal Interface Service, Staffordshire, United Kingdom

**Keywords:** Musculoskeletal, First Contact Physiotherapy, General practice, Primary care

## Abstract

**Objective:**

First Contact Physiotherapy (FCP) is a primary care model where expert musculoskeletal (MSK) physiotherapists undertake the first patient consultation, to enhance MSK-patient care and free-up GP capacity. The authors report the qualitative findings from the FCP National Evaluation (Phase 3) which evaluated the FCP model against pre-agreed success criteria.

**Design and setting:**

A mixed-methods 24-month service evaluation involving FCP sites across England.

**Methods:**

Data were collected at 2 time points, year 1 and year 2. Data were collected using individual interviews and focus groups, transcribed verbatim and analysed using a hybrid inductive and deductive thematic analysis. Participants were recruited from all stakeholder groups; patients, physiotherapists, general practitioners and administration staff.

**Results:**

A total of 6 sites were recruited over both rounds of data collection demonstrating a wide range of service models. Thirty-nine participants were recruited including fourteen patients. All six qualitative success criteria were met. GPs’ discourse reflected confidence in the FCP service and competence of the FCPs. Patient discourse reflected self-efficacy and confidence in self-management techniques and reported FCP as a positive experience. FCPs saw providing advice about work related issues as integral to their role and patient discourse reflected perceived benefit from the advice offered. Staff discourse reflected a positive experience of working with, and in, the FCP services.

**Conclusion:**

Ahead of the planned scale-up of the FCP primary care model across the UK, this evaluation provides useful insights and recommendations to facilitate successful FCP implementation in terms of patient outcome and experience, and staff experience.

## Introduction

MSK conditions are the leading cause of disability adjusted life years [1] and in the UK, they account for approximately 14% of General Practitioner (GP) consultations [2]. Meeting the demand for musculoskeletal (MSK) healthcare is a significant challenge and part of proposed solutions is first contact physiotherapists (FCPs). These are autonomous practitioners who have been introduced to streamline MSK care pathways, provide faster access to specialist MSK healthcare, and release GPs’ MSK capacity to manage other patients. FCPs are experienced MSK clinicians with advanced clinical practice skills able to assess, diagnose and manage the full spectrum of MSK patients, including complex cases [3].

First Contact Physiotherapy (FCP) is a primary care model that provides patients with direct access to physiotherapists, most commonly for the assessment and management of musculoskeletal disorders (MSK), without the need for a prior assessment or referral from a GP. The GP contract [3] pledges central government funding to primary care networks (groups of general practices working together) throughout the UK for the implementation of FCP services.

A small body of literature has shown value of the FCP model of care in the UK [4], [5], [6]. This is supported by several pragmatic service evaluations which all demonstrate similar positive findings albeit these were mostly small studies of individual services [4], [5], [6], [7], [8]. Positive findings included clinical effectiveness, patient and staff satisfaction and financial savings. Physiotherapists were shown to be able to appropriately manage most patients and the service appeared acceptable to patients and GPs [4], [5], [6]. Importantly, most patients were managed utilising a tailored self-management strategy within two appointments [4], [6].

Nevertheless, several more nuanced issues have been highlighted in qualitative studies of FCP services [9], [10] which suggest the need for a cultural shift from both a patient and professional perspective to enable the successful implementation of FCP. This included a shift from the patient’s perspective where they see their GP as their first contact practitioner and a shift from the physiotherapists’ perspective whereby the FCP role is seen as a reconceptualization of their traditional role, to one that mirrored the GP approach to working [9]. Since 2018, a rapid roll-out of FCP services across England was stimulated by the Elective Care Transformation Programme (ECTP) within the NHS England’s supported pilot of FCP services [11]. Services involved in the ECTP FCP pilot were required to participate in the FCP National Evaluation. The third phase of the evaluation was a national mixed-methods evaluation of the FCP model of care. Here, the authors report the qualitative findings of this Phase 3 evaluation.

### Overall aim

The overall aim of the phase 3 evaluation was to evaluate the FCP model of care against predefined service aims and success criteria.

### Objectives of the qualitative evaluation

Achieve a-priori agreement of the FCP’s service aims and success criteria, against which the new FCP model of care could be evaluated

a Describe the experiences of FCPs, GPs and general practice staff.
b Describe the role of the FCPs in providing advice about work in the context of the patient’s presenting MSK condition.
c Identify barriers to, and facilitators for, the successful implementation of the FCP model of care.
d Provide recommendations for the scalability and successful implementation of the FCP model of care.

## Methods

### Agreement of service aims and success criteria

The Evaluation team drafted a list of five service aims and 12 success criteria based on published literature and the Elective Care High Impact Interventions Implementation Framework [4], [5], [6], [7], [8], [9], [10], [11]. The draft service aims and success criteria were made available to CSP’s interactive (iCSP) FCP network, the FCP steering group and presented to a Patient and Public Involvement and Engagement (PPIE) Group for discussion and finalisation. This paper reports on six of the 12 success criteria, developed at the start of Phase 3, pertaining to the qualitative data collection (success criterion 2, 4, 7, 10, 11 and 12, shaded grey in Table 1). The development of these success criteria and the quantitative component of the evaluation (Stynes *et al*. Quantitative companion paper, 2021) addresses the remaining six criteria.

**Table 1:** Agreed service aims and success criteria.

Aim A: FCP services should reduce the workload of GPs	
Success criterion 1	The percentage of patients consulting the FCP who report (within 3 months) visiting their GP for the same problem will be: 25% or less (fully met), 26 to 50% (partially met), 51% or more (not met).

aSuccess criterion 2	GPs’ discourse reflects confidence in the FCP service and competence of the FCPs

Aim B: FCP services should provide assessment and self-management advice.	
Success criterion 3	Patients will report receiving self-management information/exercises from their FCP relating to their joint or muscle symptoms. 70% or more (fully met), 50 to 69% (partially met), 49% or less (not met).

aSuccess criterion 4	Patient discourse reflects self-efficacy and confidence in self-management techniques.

Aim C: FCP services should provide high quality care and a good patient experience to patients with MSK problems	
Success criterion 5	Patients should report improved MSK health at 3 months (as measured by achieving a minimal important change of 6 points on the MSK-HQ): 51% or more (fully met), 40 to 50% (partially met), 39% or less (not met).

Success criterion 6	Patients report being ‘Likely’ or ‘Very likely’ to recommend the FCP service to friends and family. 80% or more (fully met), 60 to 79% (partially met), 59% or less (not met).

aSuccess criterion 7	Patient discourse reflects a positive experience of FCP.

Aim D: FCP services should support patients to remain in/return to work	
Success criterion 8	Patients in employment report receiving specific advice about work. 75% or over (fully met), 50 to 74% (partially met), 49% or less (not met).

Success criterion 9	Patients will report less impact of their MSK condition on work performance at 3 months (as measured by the Stanford Presenteeism Scale): 51% or more report reduced impact (fully met), 40 to 50% (partially met), 39% or less (not met).

aSuccess criterion 10	Physiotherapist’s discourse reflects confidence in their competence to offer advice with regards to work related issues.

aSuccess criterion 11	Patient’s discourse reflects perceived benefit from the advice offered by the FCP with regards to work related issues.

Aim E: FCP services should provide staff with a positive experience	
aSuccess criterion 12	Staff discourse reflects a positive experience of working with and in the FCP services.

a Success criterion addressed by the qualitative data collection reported in this paper.

### Study design

For this qualitative investigation, data were collected *via* semi-structured interviews and focus groups. The Consolidated Criteria for Reporting Qualitative Research (COREQ) checklist [12] was used throughout to ensure necessary components of study design were reported. The published literature regarding FCP and the service aims and success criteria (Table 1) helped to provide a framework to develop the topic guides for the interviews and focus groups.

### Participant recruitment and data collection process

FCP services were invited to participate in the qualitative component *via* the interactive CSP (iCSP) network. A link FCP, usually the lead physiotherapist in the service, was identified for each participating site.

Participants were recruited in two stages (December 2018 and January to February 2020). Participant groups included patients who had attended the FCP service, FCPs, GPs, administration staff, and commissioners. A purposeful sampling method was used to recruit FCP services based on their delivery model to optimise information-rich cases [13]. Individual participants, from each participant group, were recruited by convenience. During the recruitment process the link FCP contacted all potential participants and provided them with an information sheet with all those agreeing to participate signing a consent form. A nominated code was given to each participant in order to preserve confidentiality and anonymity. Patient participants were interviewed individually to ensure each participant was allowed the opportunity to contribute fully. Thirteen of the 25 staff participants were interviewed in focus groups.

The interviews and focus groups were recorded using digital voice recorders, transcribed verbatim and coded by the evaluation team. The transcriptions were uploaded into QSR International's qualitative data analysis NVivo 11 Software.

### Data analysis

This evaluation used a hybrid deductive and inductive thematic analysis [14], [15]. The service aims and success criteria and published literature regarding FCP provided *a priori* theories that informed the deductive analysis and concurrent inductive analysis allowed the emergence of novel themes. In accordance with COREQ guidelines [12] transparency in data coding is described. All transcripts were coded by two researchers (FM, RG) with co-investigators resolving any disagreements. A total of 50% of the transcripts were joint-coded. Previous research has shown this method is adequate to demonstrate consistency in coding, interpretations and inferences made by the lead researcher [16]. Using this method demonstrated excellent agreement in coding and analysis of the data and no further joint coding was deemed necessary.

## Results

### Sites and participants in the qualitative data collection

#### Sites

In stage 1 two sites were visited. In stage 2, four new sites were visited and one site from stage 1 was revisited. The second site from stage 1 no longer delivered an FCP service. Site visits to Scotland and Northern Ireland were cancelled due to the COVID-19 pandemic. Sites varied in the number of GP practices and population serviced and the service model delivered. This ranged from a single GP practice (site 2) with a population of 10,000 to 68 GP practices with a population of 800,000 (site 6). In one site, the FCP was employed directly by the general practice (site 3). In the remainder sites, there were a number of employment models ranging from primary care FCPs employed by a GP Federation (site 5) to a secondary care outreach model where FCPs based in secondary care were delivering sessions in primary care community settings and general practice (sites 2,4 and 6). In most sites, financial support was from commissioning organisations and was frequently FCP pilot initiative funding. As a consequence of this variability in funding, the FCP sessions per site varied. On average, most GP practices had an FCP located in their practice between 0.5 to one day per week. Table 2 gives details of the FCP sites included.

**Table 2:** FCP site details for qualitative evaluation.

Site	Site 1^a^	Site 2^b^	Site 3	Site 4	Site 5	Site 6
Length of time FCP site established	6 months (in stage 1 visit)	3 months	4 years	17 months	2 years	5 years

Service model	Community care provider	Secondary care provider	Employed by GP practice	Secondary care provider	GP Federation	Secondary care provider

Population served by FCP service	50,000	10,000	19,000	160,000	290,000	800,000

Number of GP practices	5	1	1	20	37	68

FCP capacity/GP practice/week	1 day	1 day	5 days	1 day	0.5 to 1 day	0.5 to 2 days

Number of FCPs/FTE	3/1	2/0.2	1/0.75	1/0.2	8/7.45	14/14

FCP Banding/s	2 × B8a, 1 × B7	8a	8a	8a	3 × B8a, 5 × B7	7 & 8a

Advanced skills of FCPs	B8a: NMP, injection therapy. B7&B8a: radiology & pathology requesting	1 × NMP	Pending NMP & injection therapy	NMP, injection therapy, radiology requesting	NMP, injection therapy, radiology requesting	NMP, injection therapy, radiology & pathology requesting

Appointment length	20 minutes	30 minutes	20 minutes	20 minutes	30 minutes	20 minutes

a Site 1 was visited in both stage 1 and stage 2.

b Site 2 was visited in stage 1 only; FCP: first contact physiotherapist, GP: general practitioner; B: band, FTE: full time equivalent; NMP: non-medical prescriber.

### Interview and focus group participants

Over the two stages there were a total of 39 participants, this included 14 patients, 8 GPs, FCP 10 and 6 general practice administration staff. The duration of each interview and focus group ranged between 25 and 75 minutes. In total approximately 20 hours of audio data were recorded. Participant details are provided in Table 3.

**Table 3:** Participant details for qualitative evaluation.

	Data collection point 1	Data collection point 2	Data collection point 3	Data collection point 4	Data collection point 5	Data collection point 6
Site 1	GP 1	FCP 1	Patient 1 (M, White British, Age 62)	Patient 2 (M, White British, Age 53)	Patient 3 (F, White British, Age 47)	

Site 2	GP 2	FCP 2	Practice Manager 1	Patient 4 (F, British Asian, Age 62)	Patient 5 (F, White British, Age 67)	Patient 6 (M, British Asian, Age 32)

Site 3	FCP 3 + GP 3 (FG)	Practice Manager 2	Patient 7 (F, White British, Age 67)	Patient 8 (F, White British, Age 29)		

Site 4	FCP 4	GP 4	Practice Manager 3	Patient 9 (M, White British, Age 69)	Patient 10 (M, White American, Age 57)	

Site 5	FCP 5 FCP 6 (FG)	GP 5 + Practice manager (FG)	GP 6	Practice Manager 4	Patient 11 (F, White British, Age 49)	Patient 12 (F, White British, Age 75)

Site 6	FCP 7	GP 7 + Practice Manager 5(FG)				

Site 1 (revisited in stage 2)	FCP 8 FCP 9 FCP 10 + GP 8 (FG)	Patient 13 (M, White British, Age 73) Patient 14 (M, White British Age 77) (FG)	Practice Manager 6			

FG, focus group; M, male; F, female.

Evidence pertaining to the six qualitative service aims and their respective success criteria are presented below.

Success criteria 2—GPs’ discourse reflects confidence in the FCP service and competence of the FCPs.

GP participant discourse reflected confidence in the competence of FCPs, and GPs welcomed the addition of FCPs to the general practice workforce:

“It has been a fantastic addition actually. And I think I’ve seen patients who have seen [FCP] subsequently, and they’ve been delighted with the treatment that they’ve got. I think we’re very lucky for [FCP]’s experience, I mean it’s Advanced, so I don’t know how you know, how realistic that is to replicate across the whole population. Because you know, there's always going to be variability in the experience and expertise. But so far it’s been almost all positive”GP 8

The experienced FCP referenced by the GP above was representative of the FCP participants demonstrated advanced practice skills (Table 2), in line with the recommended guidelines for FCP roles [3]. The aim that FCP services should reduce the workload of GPs (service aim 1) had been an important narrative in the national FCP agenda on the basis of FCP services unburdening GPs by reducing their MSK patient workload. The stage 1 data collection had evidenced this as unclear and a complex construct. The stage 2 data collection reinforced these findings with the overriding consensus that there was currently insufficient capacity in FCP services to unburden GPs. For example, the site where 1 FCP provided 1 day of access for 20 GP practices:

“I don’t think I’ve reduced the burden on GPs in terms of, I don’t think I’ve increased their capacity, purely because I’m doing two sessions for 22 GP practices, so I don’t think they’ll notice a difference”FCP 4

When the potential to positively impact GP workload by addressing the mismatch between FCP capacity and MSK demand was explored it was viewed as a realisable objective. However, there were thought to be certain caveats. The first of these was the nature of patient complexity and comorbidity:

“Sometimes it’s appropriate to you know, when I’m seeing you know, Mr Smith about his diabetes, and he wants to talk about his knee pain you know, his osteoarthritis, then fine you know, that will always carry on.”GP 3

The second caveat related to patient beliefs and understanding, with some patients likely to default to their GP as a first point of contact: *“Well again, I think the patients have this expectation that they come and see the GP at the GP surgery”* (GP 3). However, this was acknowledged as potentially being generational: *“I think elderly patients possibly more so”* and somewhat related to a misunderstanding of physiotherapy and the FCP role: *“when you mention physio, often they’ll say oh, I don’t want someone pushing and pulling me around. And it’s saying actually, you’re not going to be pushed and pulled around, you know, this is somebody who is going to assess you, diagnose”*(GP 3).

It was acknowledged that the funding gap in existing service models needed to be addressed to release capacity and realise this objective. A practice manager (and CCG commissioner) and a GP both spoke of the ‘bravery’ required in this vision:

“I think as you said it’s sort of bravery. I think if you look at pure appointment numbers, and if every MSK issue went to the physiotherapist, then you could probably justify dropping a GP clinician. But it’s a… you've got to have the balls to do it”GP 4

Whilst the current model of FCP delivery had apparently failed to demonstrate a reduction in GP workload, the evaluation provided a novel insight into the impact of FCP on GP work experience that was seen by participants as equally important. Specifically, FCP was seen as introducing an MSK specialism into the practice. Not only was this seen as a welcome clinical resource for patients but it provided support and up-skilling to GPs and the wider general practice team. There were multiple examples of this, ranging from the informal conversations to more formal training sessions provided by FCPs:

“When we have a multidisciplinary…team meeting. Talking about complex patients, we can include those first contact practitioners. They may not be doing what they would do if they were face to face to a patient. But they can provide help and support to the whole team about understanding those areas. For instance, we’re all learning about Start Back at the moment, they can train us in Start Back”

Practice Manager 6

Success criteria 4—Patient discourse reflects self-efficacy and confidence in self-management techniques.

Success criteria 7—Patient discourse reflects a positive experience of FCP.

The service aims and success criteria 4 and 7 are reported together.

Patient discourse reflected patient expectations in consideration with the provision of advice around self-management techniques by FCPs and confidence therein. Particular reference was made to the focus on rehabilitation rather than pain relief and this was contrasted to GP care with FCP welcomed as an introduction of an MSK specialism within general practices:

“My first impression, it’s been excellent, it’s had me in and out of here way quicker than it would have done if I’d have had to wait for the GP. And actually [FCPs] knowledge and expertise is much more specific …than possibly a GP who again, I’m just stereotyping, who possibly would have focussed on the pain, and the pain relief, rather than the rehabilitation and fixing the problem. Or potentially referring me to a physio, so it’s cut that stage of it out”Patient 7

Patients spoke confidently about the self-management advice they had been given and also described an associated sense of self-efficacy:

“So it was years of, oh no, my back’s gone again, and then regretting, oh why haven’t I done my exercises? And that’s why I do the exercises [FCP] has given me. Yeah, and there's a switch, a change in how you take control of your own healthcare really”

Patient 1

Satisfaction with FCP services was reported by all patient participants. Furthermore, analysis of patient satisfaction was reported as overwhelmingly positive.

“the positiveness that we’ve had back has been quite overwhelming. 100% satisfaction, positive, excellent reviews from patient groups and patient participation”

Practice Manager 5

As seen above, inherent in the patient satisfaction expressed with FCP services was the speed and ease of access. However, this access required facilitation by GP reception/administration staff or *via* other routes (such as GP triage) as most patients were unaware of the FCP services within their general practice. In some cases, where patients were signposted to FCP following a GP consultation, it undermined the first contact principle. Patients also appeared to be cognisant of the changing face of primary care and general practice, the increasing role of administrator signposting in this evolution and how FCP sat within this contextual reconfiguration.

“I think it’s great to involve other… what do you call yourselves… practitioners, other care givers, other people who know what they’re talking about. So that the GP can see the people who are really sick and need to see the GP”Patient 7

Success criteria 10— Physiotherapists’ discourse reflects confidence in their competence to offer advice in consideration with work-related issues.

Success criteria 11—Patients’ discourse reflects perceived benefit from the advice offered by the FCP in consideration with work- related issues.

Success criteria 10 and 11 are reported together as they are closely linked. An area of particular interest for this project was that of work, and the role that FCPs play in providing employment support and advice.

All of the FCPs stated that they would consider work-related issues with every patient of working age. Of the patient participants, the majority reported discussing work and receiving advice from the FCP. The age of some patient participants meant that they were retired, however the patients who had received employment related advice found this helpful.

“So I had taken sick leave the first week when I was back, and then… So the next week she said, yes I could go but only two days a week…I must wear the sling except when I’m at my desk. So I could take it off when I’m sitting at my desk, but at all other times I have to wear the sling, so that's really helpful. And then last week it was certain exercises I had to do when I’m at my desk…and that's really helpful”

Patient 8

Two FCPs spoke specifically about using the Allied Health Professions Health and Work Report and finding this useful. However, training to use the report was patchy and inconsistent and the practical application of the form itself was described as cumbersome.

Participants from each group saw the value in FCPs being able to sign patients off work with legislative authority. Such was the strength of feeling regarding this sentiment that the current situation (no legislative authority for FCPs) was seen as strange and counterintuitive. Indeed, GPs saw FCPs as better equipped to make this assessment and described themselves as often following FCP advice. As this one GP described:

“I completely agree, I think it’s crazy … because what we do is just go on whatever the physios have deemed to be appropriate. So they say, you need a couple of weeks off we’ll just do a sick note for a couple of weeks you know. We don’t then do a whole reassessment, so actually; literally it’s just signing a form. Which it seems crazy”GP2

The only concern expressed in consideration with this came from an FCP who paralleled signing patients off work with the risks associated with other advanced practice skills:

“in terms of prescribing, I’m scared of the, maybe the addictive nature of it, you don’t know really what they’re on, or what they’re taking, and you’re being held accountable for that. But then in terms of work, if you sign someone off who, you then could get them into this cascade of being off work. So I don’t know which one scares me more”FCP 4

Nevertheless, the overwhelming consensus was that employment advice was integral to the FCP role and greater legislative accountability should be given to FCPs.

Success criteria 12—Staff discourse reflects a positive experience of working with and in the FCP services.

All the FCPs found their role a rewarding and enhancing career opportunity. For this physiotherapist it was extremely positive being at the front end of the patient journey.

“As a clinician it’s fantastic to be at the front of a pathway, to be seeing people when it’s first happening, when they’re all, even, I mean I used to work in chronic pain as well and there you’re seeing people at the very end of the pathway”FCP 5

The first contact nature of FCP work was reported as both rewarding for the physiotherapist and beneficial for patient care:

“Yeah, I love doing it. I really love working in the clinics. I feel you’re just getting to people so much quicker and giving them the right information to make changes”FCP 9

Nevertheless, the complex, unpredictable nature of this work was acknowledged as introducing some challenges and associated risk: *“You don’t know what’s coming through the door. You can’t look at your list two weeks in advance and say, oh I’ve got X, Y and Z coming in then, let’s read up on it, it’s on the day you know, you have no prior warning”* (FCP 7). Consequently, the FCPs spoke about the need to manage risk and associated competency requirements.

In both stages of data collection FCPs discussed the novelty of working in a way that emulated the GP model of care. As one FCP reported:

“I can say try these for a month and if it doesn’t work, they know that I’m in primary care… they can come back at any time. They could book an appointment with me tomorrow if they wanted to. So that reassurance for them is also quite helpful”FCP 1

Beyond GP and physiotherapy participants the benefits of FCP were widely reported by administrators and practice managers:

*“I would highly recommend the service. We’d struggle without it if you took it away”*

Practice Manager 5

FCPs were seen as highly specialised MSK practitioners. These benefits were felt to be greater when the FCP service was collocated.

## Discussion

This qualitative evaluation demonstrated success in all of the service aims and success criteria (Table 1). FCP was well received by staff and patients alike. These findings corroborate previous evidence describing the safety, acceptability and efficacy of FCP [4], [5], [6], [7], [8], [9], [10]. Furthermore, confidence in the competence of FCPs was described by GPs and patients. The FCP participants were experienced clinicians with advanced practice skills. It cannot be assumed that the findings of this evaluation apply to FCPs with less experience. In a previous evaluation, GPs were concerned that more work would be ‘bounced back’ to them with less experienced physiotherapists in FCP roles (9).

FCPs saw supporting patients to remain in/return to work as integral to their role. Patient discourse reflected perceived benefit from the advice offered by the FCP in consideration with work related issues. All participants groups advocated a greater role for FCPs in providing work advice including signing patients off, and back to, work. This echoes previous work [17] where the majority of GPs and physiotherapists who were questioned indicated that there could be benefits for patients if physiotherapists were involved in monitoring and issuing sickness certificates. A recent qualitative study exploring patient expectations of FCP [18] found that patients were seeking advice about whether, and when, they could do things, including work.

Physiotherapists described working as an FCP as a positive experience as it provided them with a rewarding career opportunity and the role gave patients timely access to their specialist care. However, some also expressed concern with the unpredictable nature of this work acknowledged as introducing some challenges and associated risk including staff burnout. This concern has also been reported elsewhere [19] with anxieties around FCPs’ wellbeing expressed both in terms of workload, diagnostic uncertainty and the subsequent risk associated with the FCP role.

Despite the widespread patient satisfaction described, there was a lack of awareness among patients about FCP services. Consequently, patient access to FCP services was on the whole reliant on signposting from reception/ administration staff. This has been previously described and could present a challenge to receptionists in terms of explaining new consultation methods to patients [9], [20], [21] and this potential unintended consequence of FCP, needs considering within the wider agenda.

Reduction of GP workload, as a result of the implementation of FCP, was not evident in the discourse of participants. The impression was that this was a result of the complex interaction of a lack of patient awareness of FCP services, limited FCP capacity and the propensity for some patients to default to their GP as the first point of contact. This complexity on the basis of the impact of FCP on GP workload has been reported elsewhere [9]. This is important because if services are built and promoted on the premise that they will reduce GP workload they will arguably fail to meet that target. It was also acknowledged by participants that the current funding models were seen as insufficient to realise the capacity increase required to genuinely impact GP workload to a measurable level. This is a pertinent finding with the current nationwide commitment to FCP whereby a population of 50,000 receives funding for 1 full time equivalent FCP [7].

The FCP service was also acknowledged as introducing an MSK specialism and a resource for GPs and the wider general practice staff. Although the introduction of FCP as an MSK specialism has been reported before [9] the potential for FCPs to support and even up skill GPs, has not. This mirrors previous evidence whereby physiotherapists provided effective undergraduate medical education for musculoskeletal system training [22]. This appears to be an important potential of the FCP role.

Strength of this evaluation is that it is the first nationwide evaluation of the FCP model of care. It is further strengthened by the multi-method approach, representation from all stakeholder groups and the wide variety of service models included. A limitation of the evaluation is that the authors were unable to collect data from some additional FCP sites in devolved nations due to travel restrictions during the Covid-19 pandemic and this somewhat limits transferability of the findings.

In conclusion our evaluation shows that the FCP model is well received and supported by all stakeholder groups. It is evident that work is evolving continuously within professional Physiotherapy networks with resources available providing guidance and advice in consideration with establishing successful FCP services. On-going evaluation and implementation research is needed to explore the most effective strategies to implement FCP services into primary care within the UK or in other countries that could promote this model of care. Future research should explore staff experiences and outcomes including satisfaction and impact on workloads for GPs, FCPs and administrators. Additionally, the cost effectiveness of the FCP models could be explored, when compared to MSK patients accessing GPs as their first point of contact.

*Ethical approval*: None required.  *Funding*: The evaluation was funded by the Chartered Society of Physiotherapy CSP Charitable Trust, Registered Charity No 279882 and The Work and Health Unit, a cross-government unit, jointly sponsored by the Department for Work and Pensions and the Department of Health and Social Care.  *Conflict of interest*: None declared.
